# Diversity of arsenite oxidase gene and arsenotrophic bacteria in arsenic affected Bangladesh soils

**DOI:** 10.1186/s13568-016-0193-0

**Published:** 2016-03-15

**Authors:** Santonu Kumar Sanyal, Taslin Jahan Mou, Ram Prosad Chakrabarty, Sirajul Hoque, M. Anwar Hossain, Munawar Sultana

**Affiliations:** Department of Microbiology, University of Dhaka, Dhaka, 1000 Bangladesh; Department of Soil, Water and Environment, University of Dhaka, Dhaka, 1000 Bangladesh; Department of Microbiology, Jessore University of Science and Technology, Jessore, 7408 Bangladesh; Department of Microbiology, Jahangirnagar University, Dhaka, Bangladesh

**Keywords:** As metabolizing bacteria, Arsenite oxidase gene (*aio*A), Arsenite resistant, Bangladesh

## Abstract

Arsenic (As) contaminated soils are enriched with arsenotrophic bacteria. The present study analyzes the microbiome and arsenotrophic genes-from As affected soil samples of Bhanga, Charvadrason and Sadarpur of Faridpur district in Bangladesh in summer (SFDSL1, 2, 3) and in winter (WFDSL1, 2, 3). Total As content of the soils was within the range of 3.24–17.8 mg/kg as per atomic absorption spectroscopy. The *aio*A gene, conferring arsenite [As (III)] oxidation, was retrieved from the soil sample, WFDSL-2, reported with As concentration of 4.9 mg/kg. Phylogenetic analysis revealed that the *aio*A genes of soil WFDSL-2 were distributed among four major phylogenetic lineages comprised of α, β, γ Proteobacteria and Archaea with a dominance of β Proteobacteria (56.67 %). An attempt to enrich As (III) metabolizing bacteria resulted 53 isolates. ARDRA (amplified ribosomal DNA restriction analysis) followed by 16S rRNA gene sequencing of the 53 soil isolates revealed that they belong to six genera; *Pseudomonas* spp., *Bacillus* spp.*, Brevibacillus* spp., *Delftia* spp., *Wohlfahrtiimonas* spp. and *Dietzia* spp. From five different genera, isolates *Delftia* sp. A2i, *Pseudomonas* sp. A3i, *W. chitiniclastica* H3f, *Dietzia* sp. H2f, *Bacillus* sp. H2k contained *ars*B gene and showed arsenite tolerance up-to 27 mM. Phenotypic As (III) oxidation potential was also confirmed with the isolates of each genus and isolate *Brevibacillus* sp. A1a showed significant As (III) transforming potential of 0.2425 mM per hour. The genetic information of bacterial arsenotrophy and arsenite oxidation added scientific information about the possible bioremediation potential of the soil isolates in Bangladesh.

**Electronic supplementary material:**

The online version of this article (doi:10.1186/s13568-016-0193-0) contains supplementary material, which is available to authorized users.

## Introduction

Arsenic (As) is one of the naturally occurring poisonous element that is widely distributed in soil, minerals, water and biota resulting from either weathering, volcanic activity, leaching or from anthropogenic activities (Cavalca et al. 2013; Mandal and Suzuki 2002). Irrigation water, if contaminated with high levels of As, may result in food chain contamination and loss of crop yield. Bangladesh is adversely affected by As contamination of groundwater. More than 60 % of the ground-water in Bangladesh contains naturally occurring As, with concentration levels often exceeding 10 μg/l which is the maximum concentration of As in drinking water recommended by WHO (Caussy and Priest 2008; Dadwhal et al. 2011). In recent decades, about 30 % of the people in Bangladesh have been exposed to health hazardous level of As from drinking water (Argos et al. 2010) while the total estimated population is about 164 million (Bureau 2010). About 75 % population in Bangladesh solely depend on groundwater for drinking purposes in rural areas, which made the situation worse. Although there is periodical monitoring of groundwater As concentration in Bangladesh, the irrigation soils are far less investigated and it is likely that the rapid spread of As might enter the irrigation soil and plant population destroying the food chain (Sultana et al. 2015). Therefore, it becomes a critical requirement to ensure As free irrigation to remediate As from soils, plants and food chain. The abundance of As and its species in the environment triggered a large number of bacteria developing various As resistance mechanisms including minimization of the uptake of As through the system for phosphate uptake, by per-oxidation reactions with membrane lipids (Cervantes et al. 1994). Microorganisms have specific enzymes or respiratory chains to mediate As redox transformations (Oremland and Stolz 2005). The oxidizing ability of microorganisms has been a recent concern for removal of As (Lievremont et al. 2003). Heterotrophic bacteria oxidize As (III) by peri-plasmic enzyme called arsenite oxidase. The autotrophic bacteria oxidize As (III) by a mechanism which reduces the oxygen and nitrogen, the energy for fixing the oxygen in the organic cellular materials is used here (Donahoe-Christiansen et al. 2004). Besides, phototrophic bacteria can oxidize As (III) during their photosynthesis (Kulp et al. 2008). These bacteria thus play an important role in As mobilization study on microbial transformation of arsenic has not so much explored in tropical country like Bangladesh. There is limited knowledge about arsenite oxidizing and resistant bacterial diversity in Bangladeshi soils and also the mechanism of arsenite oxidase, its diversity among bacterial population. According to a survey report by British Geological Survey (BGS), Faridpur is one of the worst affected districts by As contamination in ground water (BGS Technical report WC/00/19, volume 1). It is likely that As might enter the irrigation soil. However, no specific study on the As contaminated soils at Faridpur or the irrigation soils have so far been investigated. Therefore, attempts were made to survey the soils of As affected area, Faridpur targeting soil samples nearby contaminated groundwater wells both in summer and winter season to look at the variation in As content as well as to learn the diversity of As metabolizing bacteria and their respective genes.

## Materials and methods

### Collection of soil samples and geochemical analysis

Soil samples were collected from As prevalent area of Faridpur district, a previously reported As prone zone (Rasul et al. 2002). The locations selected were Bhanga, Charvadrason and Sadarpur upazilla (GPS coordinates: 23.3833°N 89.9833°E, 23.60°N 89.83°E and 23.4764°N 90.0333°E respectively). Surface soil samples (0–15 cm) were collected in two replicates from above mentioned three different location of Faridpur district both in summer season and winter season in 2013. For soil total As measurement, the samples were digested following heating block digestion procedure (Humayoun et al. 2003) and the total As concentration were estimated by atomic absorption spectrophotometer (AAS) (Perkin Elmer, Analyst 400) accompanied with hydride generation system (minimum detection limit 0.02 mg/kg). Soil pH was measured by electrometric method with the help of a pH meter using combination glass electrode. Organic carbon was measured by Walkley and Black method (minimum detection limit 0.42 %) (Walkley and Black 1934). Chloride, nitrate, phosphate, sodium and potassium content were estimated by American Public Health Association recommended method (Association APH 1992). The total nitrogen content was estimated by Kjeldahl method (Association APH 1992).

### Total soil DNA extraction and PCR

DNA from soil samples was prepared according to the modified method described previously (Bürgmann et al. 2001). At first, 0.5 g of each of the soil samples was taken in a 2 ml microcentrifuge tube (extragene, USA) and 560 µl TE buffer was added followed by addition of 6 µl (100 mg/ml) lysozyme in each tubes. Then samples were incubated at 37 °C for 1 h. 6 µl proteinase K (20 mg/ml) and 30 µl sodium dodecyl sulphate, SDS (10 % w/w) were added and the samples were incubated at 37 °C for 30 min at 50 °C. Then 100 µl 5 M NaCl and 80 µl pre-warmed 10 % Cetyltrimethyl ammonium bromide (CTAB) solution was added followed by gentle mixing and incubation at 65 °C water bath for 10 min. After processing, the tubes were centrifuged at 16,000×*g* for 5 min and an aliquot depending on the amount of soil and buffer volume of the supernatant fluid was transferred into a fresh sterile 2-ml microtube. Equal volume of choloroform: isoamayl alcohol (24:1) was added and was vortex for 1 min followed by centrifugation for 10 min. Supernatant was collected in a fresh 2 ml tube and equal volume of phenol: choloroform: isoamayl alcohol (25:24:1) was added and shaked for 1 min with centrifugation for 10 min. Supernatant was collected in a fresh 2 ml tube for further analysis. Then 0.3 volume of ammonium acetate and 0.7 volume of isopropanol was added and DNA was precipitated by gentle mixing and the mixture was centrifuged for 20 min. Pellet was dried and re-suspended with 100 µl of TE buffer. Soil DNA was subjected to PCR using universal primers for bacterial 16S rRNA gene as well as specific genes for arsenite resistance (*ars*B, *acr*3P) and arsenite oxidizing (*aio*A) (Additional file 1: Table S1).

### Detection and cloning of the *aio*A gene

Extracted DNA from soil was used as template for amplification of *aio*A gene (~1100 bp) with primers BM1-2Fand BM3-2R as previously described (Quéméneur et al. 2008) (Additional file 1: Table S1). The PCR program was as follows: 95 °C for 5 min and then 35 cycles at 95 °C for 60 s, 52 °C for 45 s and 72 °C for 90 s, followed by a 10-min extension time at 72 °C. After amplification, gel slices containing the PCR products were excised and purified using Wizard^®^ SV Gel and PCR Clean-Up System (Promega, Madison, WI, USA). PCR products were ligated into the plasmid vector pCR™4-TOPO^®^ (Invitrogen, USA) and then cloned into *Escherichia coli* DH5α in accordance with the manufacturer’s instructions. Transformants were grown on LB agar containing Kanamycin (100 μg/ml) and positive clones were confirmed for the right insert using PCR with primers (T3 and T7), which were complementary to the flanking regions of the PCR insertion site of the pCRTM4-TOPO^®^ vector. All clones containing inserts of the correct size were stored in LB medium at −20 °C. The PCR products were digested with restriction endonucleases *Alu*I, at 37 °C for 4 h. The restriction enzyme digests were separated on a 1 % agarose gel running in 1× TAE buffer at 100 V for approximately 1 h. Fragments shorter than 80 bp were not taken into consideration, because they were very close to the detection threshold. According to restriction fragment length polymorphism analysis patterns, clones were grouped into RFLP groups.

### Sequence alignment and phylogenetic analysis of *aio*A gene

The PCR amplified products of cloned *aio*A genes were purified using the Wizard PCR SV Gel and PCR Clean-Up System kit (Promega, USA). RFLP group representative purified PCR products were sequenced by ABI sequencer (ABI Prism 3130 Genetic Analyzer, USA) using forwards T3 and reverse T7 primers. Partial *aio*A gene sequences were subjected to BLASTN analysis (http://www.ncbi.nlm.nih.gov/) to identify the species exhibiting the most significant homologies. The nucleotide sequences of the As-metabolizing bacterial strains have been deposited in GenBank, and Phylogenetic trees of translated AioA amino acid sequences were generated using the neighbour-joining algorithms following the p-distance model in (Tamura et al. 2007). The level of support for the phylogenies, derived from neighbour-joining analysis, was determined from 1000 bootstrap replicates. The phylogenetic tree was drawn to scale with branch lengths shown in the same units as for inferred evolutionary distances.

### Enrichment of arsenite metabolizing bacteria

Approximately 6 g of each of the soil samples were enriched in 60 mL of minimal salt medium (MSM) containing 2 mM of sodium arsenite, NaAsO_2_ (Merck, Germany) for recovery of chemolithoautotrophic As(III) oxidizing bacteria (Santini et al. 2000; Sultana et al. 2012). In parallel, heterotrophic enrichment medium was used for As resistant bacteria (Gihring and Banfield 2001). Additionally, cycloheximide (80 mg/l) was added to exclude any fungal growth in the enrichment media (Sultana et al. 2011). All the enrichment broths were incubated aerobically on a rotary shaker at 30 °C and 120 rpm. Periodically after 4 weeks of incubation, 10 ml of each of the enrichments was transferred to a 250-ml Erlenmeyer flask containing 50 ml of the respective enrichment medium incubated on a rotary shaker at 30 °C and 120 rpm. For isolation, 10^3^-fold dilution of each of the soil samples was done and was spread on solid media plates of autotrophic and heterotrophic growth. Secondly, enrichments were either serially diluted and spread or directly streaked onto minimal salts enrichment agar [2 % (w/v)] medium containing arsenite (2 mM). After growth, a number of different colonies were selected, purified, and kept for further studies.

### PCR of 16S rRNA and functional arsenotrophic genes within the Isolates

Bacterial DNA was extracted with ATP™ Genomic DNA Mini Kit (ATP Biotech Inc, USA) according to the kit manual. PCR using universal primers for bacterial 16S rRNA gene, arsenite resistant (*ars*B, *acr*3P) and oxidizing genes (*aio*A) was done. The PCR reaction mixture was prepared by mixing the components at given volumes described in Additional file 1: Table S1.

### Amplified Ribosomal DNA Restriction Analysis (ARDRA) and bacterial identification

Complete digestion of 16S rRNA genes of the As resistant isolates was done using the *Alu*I (Promega, USA) restriction enzyme. The restriction mixes (20 μl of final volume) were carried out for 4 h at 37 °C. Each reaction tube contained 2 μl of 10X incubation buffer, 0.2 μl of bovine serum albumin, 6 U of the restriction enzyme, 2.5 μl of distilled water and 15 μl of PCR product. Each ARDRA group specific isolates 16S rRNA gene PCR products were purified and maintained at 4 °C and sequencing was performed. Partial 16S rRNA gene sequences were subjected to BLASTN analysis (http://www.ncbi.nlm.nih.gov/) to identify the species exhibiting the most significant homologies. The nucleotide sequences of the As-metabolizing bacterial strains have been deposited in GenBank, and Phylogenetic trees of partial 16S rRNA gene sequences were generated using the neighbour-joining algorithms following the p-distance model in Mega 4. Determination of minimum inhibitory concentration (MIC).

On the basis of ARDRA genotyping pattern, representative isolates of specific groups were selected to determine the level of MIC of As (III). Each well of a 96 well microtiter plate was filled with 130 μl sterile autotrophic and heterotrophic broth medium and supplemented with different concentrations of As(III) as NaAsO_2_ (0–30 mM) Strains were grown in 5 ml of their respective autotrophic and heterotrophic broth medium without arsenic for 48–72 h at 28 ± 2 °C on a rotary shaker (150 rpm). Twenty micro-liters of bacterial inoculums (OD_590_ = 0.1) was placed in each respective well. Initial cell density and bacterial growth every after 24 h were measured using Microplate Reader (Poweam WHY101) at 590 nm.

### Phenotypic detection of arsenite oxidation

All bacterial isolates from autotrophic and heterotrophic enrichments were primarily screened for their abilities to oxidize As (III) using a qualitative KMnO_4_ screening method as described previously (Fan et al. 2008). The verification of the transforming potential of the isolated bacteria was carried out by the AgNO_3_ method (Chitpirom et al. 2009).

### Determination of arsenite oxidation efficiency of the isolates

The As (III) oxidation of phenotypically and genotypically screened randomly selected bacteria was determined using the molybdenum blue method to measure the arsenate quantum (Lenoble et al. 2003). Arsenate reacts with MoO_4_– to form an arsenate-molybdate complex. This complex reacts with ascorbic acid to produce a blue color liquid that is measured at 846 nm using spectrophotometer. A single colony of the tested bacterium was inoculated to 100 ml of MSM medium containing 125 mg/l of Na-arsenite and incubated at 27 °C with rigorous shaking. Five ml of the culture was taken every hour and mixed with Na_3_MoO_4_. A standard curve with arsenate concentration from 0 to 1.33 mM was used. In parallel the growth curve of the isolate was analyzed.

All the sequences obtained in this study have been deposited in NCBI GenBank database under the accession numbers KT835025-KT835047.

## Results

### Geochemical characterization of As contaminated soils

**Fig. 1:**
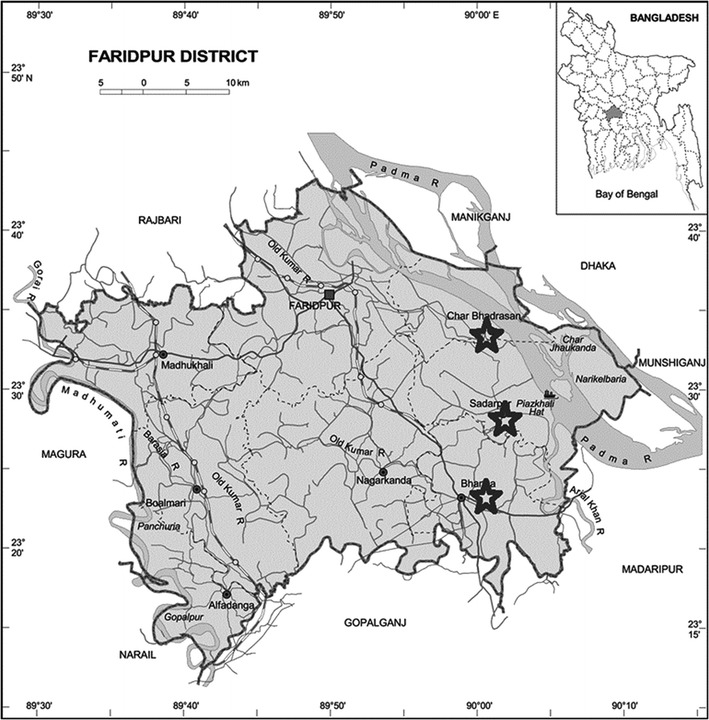
Location of three sample collection areas in Faridpur, as indicated by *asterisks* within the map

A total of six soil samples at summer season (SFDSL 1, 2, 3) and at winter season (WFDSL 1, 2, 3) from three different locations of Faridpur, Bangladesh were collected (Fig. 1). The As content of SFDSL-3 was detected to be 6.3 mg/kg in summer but was below the standard limit of 4.99 mg/kg in winter (Duxbury and Zavala 2005) (Table 1). The As content of the Charvadrason soil remained unchanged regardless to the seasonal change. The pH of the soil samples were alkaline (average pH = 8.58), organic carbon content was in the range of 4.1–5.3 and chloride content was in between 0.03 and 0.2 mg/kg. Sample WFDSL-1 contained the highest amount of chloride whereas its phosphate and total nitrogen content was low. Sample SFDSL-1 contained the highest amount of As of 17.8 mg/kg. Soil Sample SFDSL-2, collected in summer, contained the highest amount of phosphate whereas the sample collected from the same location in winter (WFDSL-2) showed almost halfway reduction of phosphate concentration.

**Table 1:** Geochemical characteristics of arsenic contaminated soil samples from Faridpur, Bangladesh (SFDSL = summer Faridpur soil, WFDSL = winter Faridpur soil)

Sample ID	Sampling site	pH	Arsenic (mg/kg)	Organic carbon (%)	Chloride (mg/kg)	Phosphate (mg/kg)	Sodium (mg/kg)	Potassium (mg/kg)	Total nitrogen (%)
SFDSL-1	Bhanga	8.67	17.8	5.1	0.1	0.178	10	12	1.0
SFDSL-2	Charvadrason	8.55	4.9	4.1	0.1	0.239	10	13	1.52
SFDSL-3	Sadarpur	8.54	6.3	4.6	0.06	0.123	12	15	2.2
WFDSL-1	Bhanga	8.59	4.6	5.3	0.2	0.110	11	13	0.92
WFDSL-2	Charvadrason	8.52	4.9	4.5	0.03	0.102	13	17	1.48
WFDSL-3	Sadarpur	8.65	3.2	4.7	0.07	0.065	12	24	2.5

### Presence of arsenotrophic genes and diversity of arsenite oxidase within soil samples

Total DNA was extracted from As contaminated soil samples and occurrence of *ars*B, *acr*3P and *aio*A gene was detected by PCR. Among the six soil samples, four (SFDSL-2, 3; WFDSL-2, 3) showed the presence of arsenical pump membrane protein specific gene *ars*B and only one soil sample (WFDSL-2) showed presence of arsenite oxidase specific (*aio*A) gene (Additional file 1: Fig. S1b-c).

**Fig. 2:**
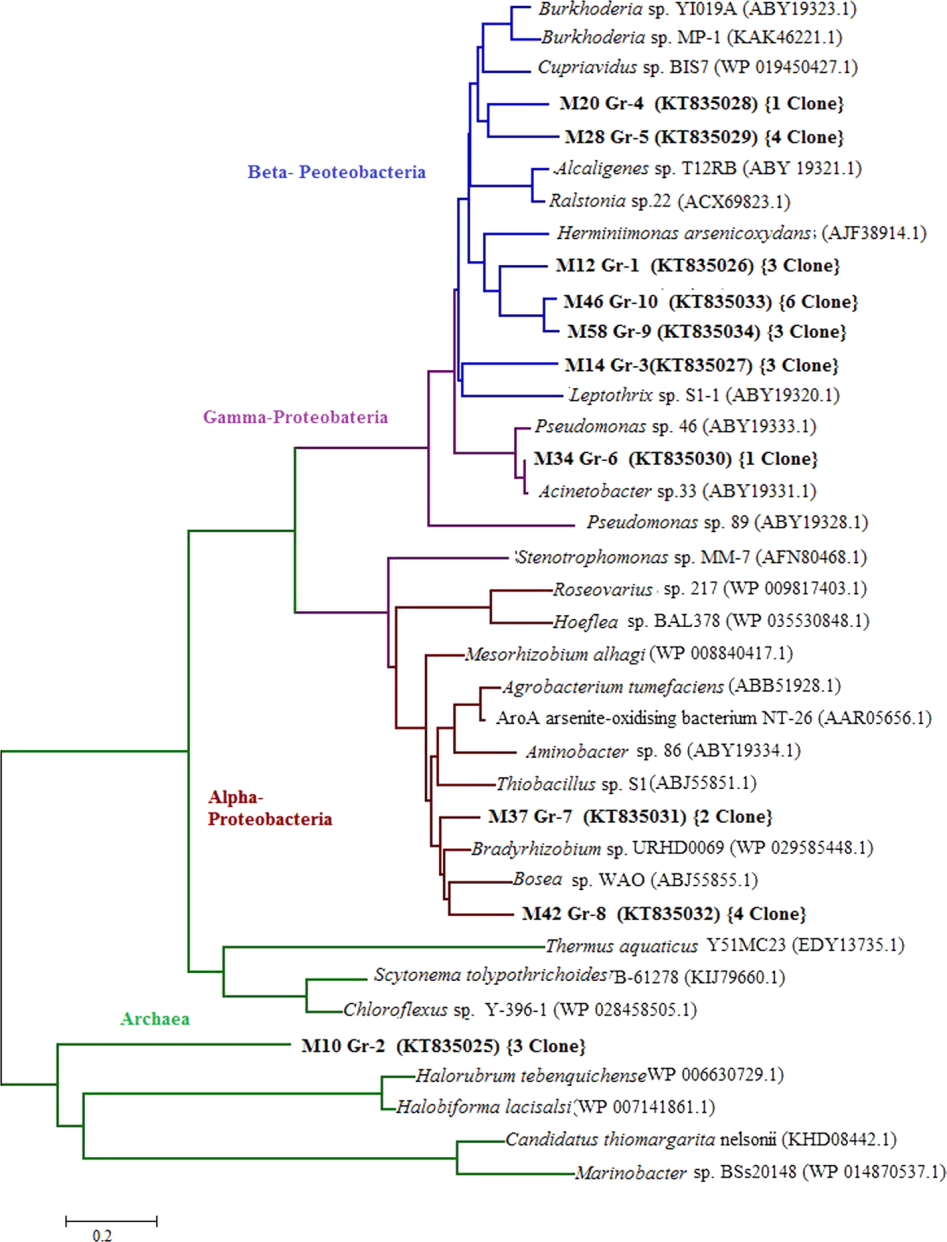
Phylogenetic tree of arsenite oxidase amino acid sequences (*bold*) obtained from the arsenite oxidase (*aio*A) clone library of arsenic affected soil W2. The tree was calculated from deduced amino acid sequences aligned in program ClaustalX and was generated in program MEGA 5 using the neighbour-joining algorithm. Bootstrap value (n = 1000 replicates)

The amplified *aio*A gene was cloned for preparation of clone library and analysis of its diversity. Thirty transformants were screened positive with PCR using primers specific for vector (T3 and T7) and arsenite oxidase gene (*aio*A).Individual clones were distributed to ten diverse RFLP groups after restriction digestion with *Alu*I enzyme of the approximate 1100 bp amplified fragment of *aio*A gene. The clone diversity was confirmed by sequencing and phylogenetic analysis of arsenite oxidase amino acid sequence (AioA). From the phylogeny reconstruction of the representative RFLP group specific clones of *aio*A gene sequences with other previously deposited sequences, it is evident that *aio*A genes were of four distinct phylogenetic lineages comprised of α, β, γ Proteobacteria and Archaea (Fig. 2). The dominant genotypic groups (Gr-10, Gr-1 and Gr-9) comprise of 12 clones were phylogenetically related to *Herminiimonas arsenicoxydans* arsenite oxidase gene specific protein. Another two RFLP groups 4 and 5 contained five clones in each group and the phylogenetic tree of these group representative clones showed their close proximity with *Cupriavidus* sp. BIS7 arsenite oxidase protein with 75 % identity. Among 30 transformants, 17 seventeen (56.67 %) were phylogenetically related to arsenite oxidase genes of β-Proteobacteria. Only one clone (M34) of group-6 was closely related to γ-Proteobacterial (*Acinetobacter* sp.) arsenite oxidase gene. A total of six clones representing RFLP group 7 and 8 showed close proximity to the reference sequence of arsenite oxidase genes of α-Proteobacteria. AioA sequence of one clone, M10, from RFLP group-2 (3 clones/10 %) formed cluster with arsenite oxidase gene sequences of archaeal origin (Fig. 2).

### Isolation and genotyping of arsenotrophic bacteria of Bangladesh soil samples

Arsenic contaminated soil samples were enriched and diluted before plating on both autotrophic and heterotrophic media supplemented with As (III). A total of 53 isolates were retrieved from the soil samples of which 29 isolates were from autotrophic and 24 were from heterotrophic growth media. These isolates were selected according to their distinguished colony morphology and Gram staining property.

**Fig. 3:**
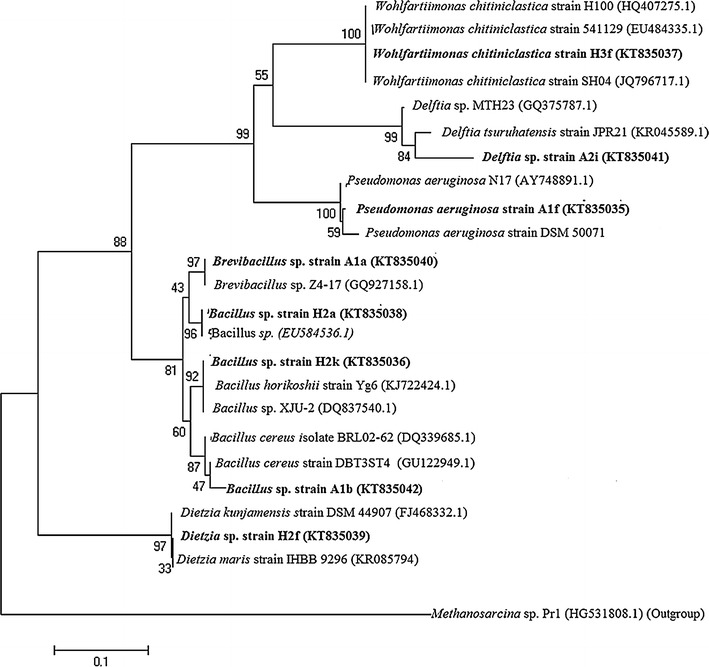
Phylogenetic tree of 16S rRNA gene sequences of arsenite resistant isolates from soil and close relative reference isolates retrieved from database with accession numbers. The tree was generated in program MEGA 5 using the neighbour-joining algorithm with the *Methanosarcina* sp. sequence serving as out-group. Bootstrap values (n = 1000 replicates) are shown at branch nodes and the *scale bar* represents the number of changes per nucleotide position

The 53 enriched isolates were distinguished into eight ARDRA groups. 16S rRNA gene sequences (Additional file 1: Fig. S2; Table 2) were analysed for their phylogenetic correlation to the nearest species level. From eight groups, the isolates randomly selected for sequence analysis were A1b, A1f, A2i, H2k, H3f, A1a, H2a and H2f. Sequencing and phylogenetic analyses distributed the 53 soil isolates into six genera belonging to *Pseudomonas* spp., *Bacillus* spp.*, Brevibacillus* spp., *Delftia* spp., *Wohlfahrtiimonas* spp. and *Dietzia* spp. (Fig. 3).

**Table 2:** Maximum identity profile of 16S rRNA gene sequences of Arsenite resistant isolates of eight genotypes of arsenite tolerant isolates according to BLAST identification

Genotypes (ARDRA); isolate number (ID)	Isolate sequenced (accession number)	Close similarity to (accession numbers), % identity	Functional gene PCR	
			*ars*B	*acr*3P
*Group 1*–*17* (A1d,A1e,A1f,A1i, A2a,A2b,A2e,A2f,A2g,A2k, A3b,A3d,A3e,A3g, A3h,A3i,A3j)	A1f (KT835035)	*Pseudomonas aeruginosa* N17, 99 %	A2f, A2g,A2k, A3b,A3d,A3e,A3g,A3h,A3i,A3j.	A1d,A1f, A1e, A1i, A2b, A2f, A2g, A2i, A2k, A3b,A3hA3i, A3j
*Group 2*; *13* (A2h,A2i,A2j,A2l,A3a,A3k,A3l,H2i,H3h,H3n,H3c,H3g,H3a)	A2i (KT835041)	*Delftia* sp., 95 %	A2i,	
*Group 3*; *6* (H2k,H3m,H3o,H3q,H3b,H3e)	H2k (KT835035)	*Bacillus* sp. XJU-2; 100 %	H3o	
*Group 4*; *4* (A2c,A2d,A3f,H3f)	H3f (KT835037)	*Wohlfahrtiimonas chitiniclastica* strain H100, 100 %	H3f	
*Group 5*; *7* (H1a,H1b,H1c,H1d,H2a,H2e,H2b)	H2a (KT835038)	*Bacillus* sp. cp-h20, 100 %		
*Gorup 6*; *1* (H2f)	H2f (KT835036)	*Dietzia* sp. WR-3, 99 %		
*Group 7*; *1* (A1a)	A1a (KT835040)	*Brevibacillus* sp. ABR4, 99 %		
*Group 8*; 4 (A1b,H3i,H3j,H3k)	A1b KT835042)	*B. cereus* strain FM-4, 99 %	A1b, H3k

**Fig. 4:**
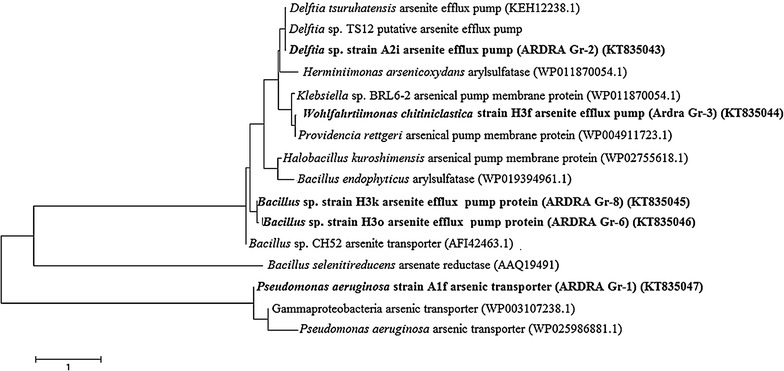
Phylogenetic tree of arsenical pump membrane protein genes (*ars*B*, acr*3P) obtained from arsenite resistant isolates from soil samples. The tree was calculated from deduced amino acid sequences aligned in program ClaustalX and was generated in program MEGA 5 using the neighbour-joining algorithm. Bootstrap value (n = 1000 replicates)

Arsenite efflux pump protein gene *ars*B was detected within the genera *Pseudomonas*, *Delftia*, *Bacillus* and *Wohlfahrtiimonas* whereas the arsenic transporter protein gene *acr*3P was only retrieve in *Pseudomonas* (Table 2; Fig. 4).

### Determination of MIC of As (III)

**Fig. 5:**
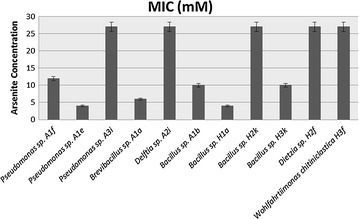
Minimum inhibitory concentration of arsenite in ARDRA group specific soil isolates. **a** Autotrophic isolates [Group-1: *Pseudomonas aeruginosa* (A1f, A1e, A3i) G-2: *Delftia* sp. A2i, G-7: *Brevibacillus* sp. A1a, G-8: *Bacillus cereus* A1b]. **b** Heterotrophic isolates [G-5: *Bacillus* sp. H1a, G-6: Dietzia sp H2f, G-3: *Bacillus* sp. (H2k, H3e), G-8: *B. cereus* H3k, G-4: *Wohlfahrtiimonas chitiniclastica* H3f]

The MICs of isolates representing each of the 8 ARDRA groups and six individual genera were ranged from 4 to 27 mM (Fig. 5). The highest MIC of 27 mM was exhibited by five isolates; *Pseudomonas* sp. A3i from ARDRA group 1, *Delftia* sp. A2i from ARDRA group 2, *Bacillus* sp. H2k from ARDRA group 3, *Wohlfahrtiimonas chitiniclastica* H3f from ARDRA group 4 and *Dietzia* sp. H2f from ARDRA group 6. All five isolates contained the arsenite efflux pump specific gene *ars*B (Table 2). Two isolates of *Bacillus cereus* A1b and H3k (both *ars*B containing) chosen from ARDRA group-8 were inhibited in the presence of at least 10 mM of arsenite. The MIC of As (III) for ARDRA group 5 and 7 representative isolates *Bacillus* sp. H1a and *Brevibacillus* sp.A1a were 4 and 6 mM respectively (Fig. 5).

### Phenotypic screening and quantitative determination of arsenite oxidation

Ten isolates (A1a, A1b, A1i, A1f, A2a, A2b, A2d, H1a, H2k, and H2f); seven autotrophic and three heterotrophic isolates from soil were primarily screened as As (III) oxidizing by AgNO_3_ and KMnO_4_ phenotypic assay. But all of these isolates were PCR negative.

Molybdenum blue assay was done to detect the true potential of an isolate to transform toxic form of arsenic. As (III) oxidation potential of three isolates designated as *Bacillus* sp. A1b, *Pseudomonas aeruginosa* A1i and A2a were determined as they were screened As (III) oxidizing by phenotypic (KMnO_4_ and AgNO_3_) test. Additionally, another isolate *Brevibacillus* sp. A1a was taken which was phenotypically positive for arsenite oxidation. Here the minimal salt media) was used for growth and oxidation of the isolates. All of the isolates showed fairly high potential to oxidize and thus detoxify As (III). The experiment was repeated twice to confirm the reproducibility. The isolates were able to oxidize arsenite to arsenate aerobically. The arsenite oxidation started 3 h after inoculation in case of *Bacillus* sp. A1b. *Brevibacillus* sp. A1a and *Pseudomonas* sp. A1i and after 4 h, 100 % of Na-arsenite was oxidized to arsenate. Finally, the oxidation rate for isolate A1a, A1b, A1i and A2a were 0.2425, 0.16, 0.138 and 0.194 mM As (III) per hour during their log phase respectively (Additional file 1: Fig. S5).

## Discussion

Arsenic contamination in soil and groundwater in Bangladesh surpasses any incident seen before. Irrigation water in Bangladesh containing high level of As which may result in food chain contamination and loss of crop yield (Anawar et al. 2002). The high level of As in groundwater as well as surrounding soil environments in the region of South East Asia, especially in Bangladesh have caused serious public health concern (Watanabe et al. 2004). Among all arsenic species, arsenite is more mobile, highly soluble and more toxic. The best approach to minimize arsenite contamination is to oxidize it into less toxic arsenate which is less soluble and can easily be removed (Lim et al. 2014). The present study was designed to analyze the diversity of arsenite oxidase gene, necessary for biotransformation of arsenite to arsenate and isolation of the arsenite metabolizing bacteria from arsenic contaminated soil of Faridpur, Bangladesh.

### Diversity of bacterial arsenite oxidase (*aio*A) gene in arsenic contaminated soil

Microbial oxidation of arsenite is a critical link in the global As cycle and is mediated by the functional gene of arsenite oxidase (*aio*A). Phylogenetically diverse arsenite-oxidase gene have been detected in a number of species; *Achromobacter* sp., *Pseudomonas* sp., *Alcaligenes faecalis*, *Thiobacillus ferrooxydans*, and *T. acidophilus* from various aquatic and soil environments (Inskeep et al. 2007). The present investigation is one of the first endeavors to detect the diversity of arsenite oxidase gene in Bangladeshi soils. Therefore, the total DNA of Faridpur soils was amplified for arsenite oxidase gene (1100 bp) and could only retrieve it from Charvadrasan soil at winter season (WFDSL-2). Cloning and sequence analysis revealed the genetic diversity of arsenite oxidase gene in soil sample WFDSL-2. Various studies reported that the branching of the *aio*A follows the 16S rRNA gene based phylogenetic lineages indicating the ancient origin of this enzyme (Quéméneur et al. 2008). So our analysis was well corroborated with this principle. The AioA amino acid structure usually possess a conserved catalytic motif (IHNRPAYNSE) with some inconsistencies reported for *aio*A of several species showing divergence from the major taxonomic position such as *Thermus* sp., *Halorubrum* sp. (Sultana et al. 2012). In this study, nine clones showed its conserved catalytic regions in amino acid sequence alignment (Additional file 1: Fig. S4) but RFLP group-2 representative clone M10 amino acid sequence alignment showed divergence in its conserve catalytic domain and was clustered with arsenite oxidase gene sequence of archaeal origin.

### Diversity of arsenotrophic bacteria and their functional genes determinants

One of the focuses of this study was to enrich and isolate arsenotrophic bacteria (both arsenite tolerant and arsenite transforming) from soils for analysis of their distribution and diversity with different levels of arsenic contaminated samples. The predominant gram positive genotypes were phylogenetically associated with *Bacillus* spp. and *Brevibacillus* spp. and the predominant gram negative genotypes were dominated with *Pseudomonas* spp. followed by, *Delftia* spp. *Wohlfahrtiimonas* sp. and *Dietzia* spp. Failure in detection of arsenite oxidizing gene in bacteria might be due to mutation in primer binding site (Sultana et al. 2012) or requirements of specific nutrient supplements for their growth. Das et al. 1996 reported that *Bosea* sp. May lack ribulose 1,5-bisphosphate carboxylase, a key enzyme of carbon dioxide fixation and glutamate dehydrogenase activity of this genus may be insufficient for ammonia assimilation (Das et al. 1996). So, it was evident that lack of cultivation of such species from soil was due to inefficient nutrient supplements.

The study also investigated the functional affiliation of arsenite tolerance of the bacteria within arsenotrophic bacterial community. Resistance to arsenic species in both isolate gram-positive and negative bacteria results from energy-dependent efflux of either arsenate or arsenite from the cell, mediated through the *ars* operon (Cervantes et al. 1994; Sanyal et al. 2014). Sixteen *ars*B positives isolates were screened from the soil environment which was in agreement with a report by Achour et al. (2007). We also detected the coexistence of any two types of arsenite transporter genes within the same strain such as *Pseudomonas* sp.(ARDRA Group -1 representative isolates) such as A2f, A2g, A2k, A3b, A3i, A3h, A3j; *Delftia* sp. (ARDRA Group-2) representative A2i, contain both *ars*B and *acr*3P(2). Furthermore, a concrete correlation was obtained between the MICs of the arsenite and presence of *ars*B gene within the isolates.

### Arsenite transforming isolates and their transformation efficiency

Both phenotypic (KMnO_4_, AgNO_3_) and genotypic (*aio*A gene PCR) methods were employed in this study to screen for arsenite transforming bacteria. Initially according to KMnO_4_ and AgNO_3_ tests, ten isolates were considered as arsenite oxidizing, but in functional gene PCR, none of this isolates were arsenite oxidase gene *aio*A positive. The reason could be the presence of novel arsenite oxidase gene within them (Sultana et al. 2012) or mutation in primer binding sites (divergence in its conserved catalytic domain) of the isolates. It was later proved by the quantitative assay of arsenite oxidation in case of isolated *Brevibacillus* sp. A1a. The bacteria was phenotypically potential for arsenite oxidation but was genotypically failed to amplify with *aio*A specific primers. The arsenite oxidation potential of *Brevibacillus* sp. A1a exhibited the arsenite oxidation rate of 0.2425 mM sodium arsenite per hour in aerobic condition. It was recently reported that, an As resistant *Brevibacillus* sp. which could resist As(III) of 17 mM was reported having arsenic removal capacity under aerobic culture conditions (Mallick et al. 2014).

Bioremediation of arsenic contaminated soils and groundwater shows a great potential for future development due to its environmental compatibility and possible cost effectiveness. Biological methods involving microbial activities can be used efficiently to treat arsenic (Wang and Zhao 2009). Although the microbiological study of As is almost a century old, we still have only a limited understanding of the ancient processes and complexities by which prokaryotes utilize or tolerate As. Diversity of arsenic metabolizing bacteria are insightful to learn the geological cycling of metals and metalloids within the soil habitat. The gene diversity allow us to predict novel functionality and presence of unusual As transforming indigenous bacterial community. Moreover, complete genetic information and transformation potential of individual isolates might help to select any metal accumulating species to alleviate soil As content. That will further guide us to construct genetically engineered bacteria with potential gene construct or using respective enzyme system to develop a sustainable strategy at specific contaminated sites. We believe this work investigated the genetic information of indigenous bacterial arsenotrophy and arsenite oxidation necessary for bioremediation potential of these soil isolates in Bangladesh.

## Additional file

10.1186/s13568-016-0193-0 Primer sequences used for detection of bacterial 16S rRNA gene, arsenite resistance and oxidizing genes, and corresponding annealing temperature used for PCR. **Fig. S1.** PCR specific amplicon of 16S rRNA (a), arsenite resistance gene arsB (b) and arsenite oxidizing gene aoxB (c) of soil total DNA. **Fig. S2.** RFLP groups using AluI restriction digestion of *aioA* gene containing clones. **Fig. S3.** Different fragments obtained from Alu1 enzyme digestion of the 16S rRNA gene PCR product (approx. 1400–1450 bp) of arsenite resistant isolates. Representative groups are shown here. Uncut experimental DNA incubated under same condition was used as control. Marker used was 1 kb and 100 bp. **Fig. S4.** Amino acid sequence alignment (MEGA 5) of ten RFLP groups representative cloned arsenite oxidase genes from WFDSL-2 soil sample showing conserved catalytic motif. **Fig. S5.** Growth kinetics and corresponding oxidation of arsenite, As (III) to arsenate, As (V) by isolate (a) A1a (b) A1i.
