# Supplementary material for: Diversity of arsenite oxidase gene and arsenotrophic bacteria in arsenic affected Bangladesh soils
Source: AMB Express. 2016 Mar 15;6:21. doi: 10.1186/s13568-016-0193-0 (PMC4792827; doi:10.1186/s13568-016-0193-0)
Supplement: Supplementary file 1 — 10.1186/s13568-016-0193-0 Primer sequences used for detection of bacterial 16S rRNA gene, arsenite resistance and oxidizing genes, and corresponding annealing temperature used for PCR. Fig. S1. PCR specific amplicon of 16S rRNA (a), arsenite resistance gene arsB (b) and arsenite oxidizing gene aoxB (c) of soil total DNA. Fig. S2. RFLP groups using AluI restriction digestion of aioA gene containing clones. Fig. S3. Different fragments obtained from Alu1 enzyme digestion of the 16S rRNA gene PCR product (approx. 1400–1450 bp) of arsenite resistant isolates. Representative groups are shown here. Uncut experimental DNA incubated under same condition was used as control. Marker used was 1 kb and 100 bp. Fig. S4. Amino acid sequence alignment (MEGA 5) of ten RFLP groups representative cloned arsenite oxidase genes from WFDSL-2 soil sample showing conserved catalytic motif. Fig. S5. Growth kinetics and corresponding oxidation of arsenite, As (III) to arsenate, As (V) by isolate (a) A1a (b) A1i. [file 13568_2016_193_MOESM1_ESM.pdf]

Table S1: Primer sequences used for detection of bacterial 16S rRNA gene, arsenite resistance and oxidizing genes, and corresponding annealing temperature used for PCR

Target gene	Primers	Sequences (5′→ 3′)	Amplicon size (bp)	Annealing T (^{0}C)	Reference
16S rRNA	27F	5’AGAGTTTGATCCTGGCTCAG-3’	~1400-1450	55	Lane *et al.*, 1991
	1492R	5’-GGTTACCTTGTTACGACTT-3’			
*aio*A	AOX-F-A2	5’TGC ATC GTC GGC TGY GGN TAY3’	670	57	Sultana *et al.*, 2012
	AOX-R-E2	TTC GGA GTT ATA GGC CGG NCK RTT RTG			
	aoxBM1-2F<br>aoxBM3-2R	5’CACTTCTGCATCGTGGGNTGYGGNTA-3’<br>5’-TGTCGTTGCCCCAGATGADNCCYTTYTC-3’	1100	52	QUEMENEUR ET AL.,2008
arsB	darsB1F	5’GGTGTGGAACATCGTCTGGAAYGCNAC-3’	750	55	Achour *et a,l.*, 2007
	darsB1R	5’CAGGCCGTACACCACCAGRTACATNCC-3’			
*acr*3P(1)	dacr1F<br>dacr1R	5’GCCATCGGCCTGATCGTNATGATGTAYCC3’<br>5’CGGCGATGGCCAGCTCYAAYTTYTT 3’	750	55	Achour *et a.l.*, 2007
Topo TA	T3	5' ATTAACCCTCACTAAAG 3'	Varies	48	
	T7	5' AATACGACTCACTATAG 3'			

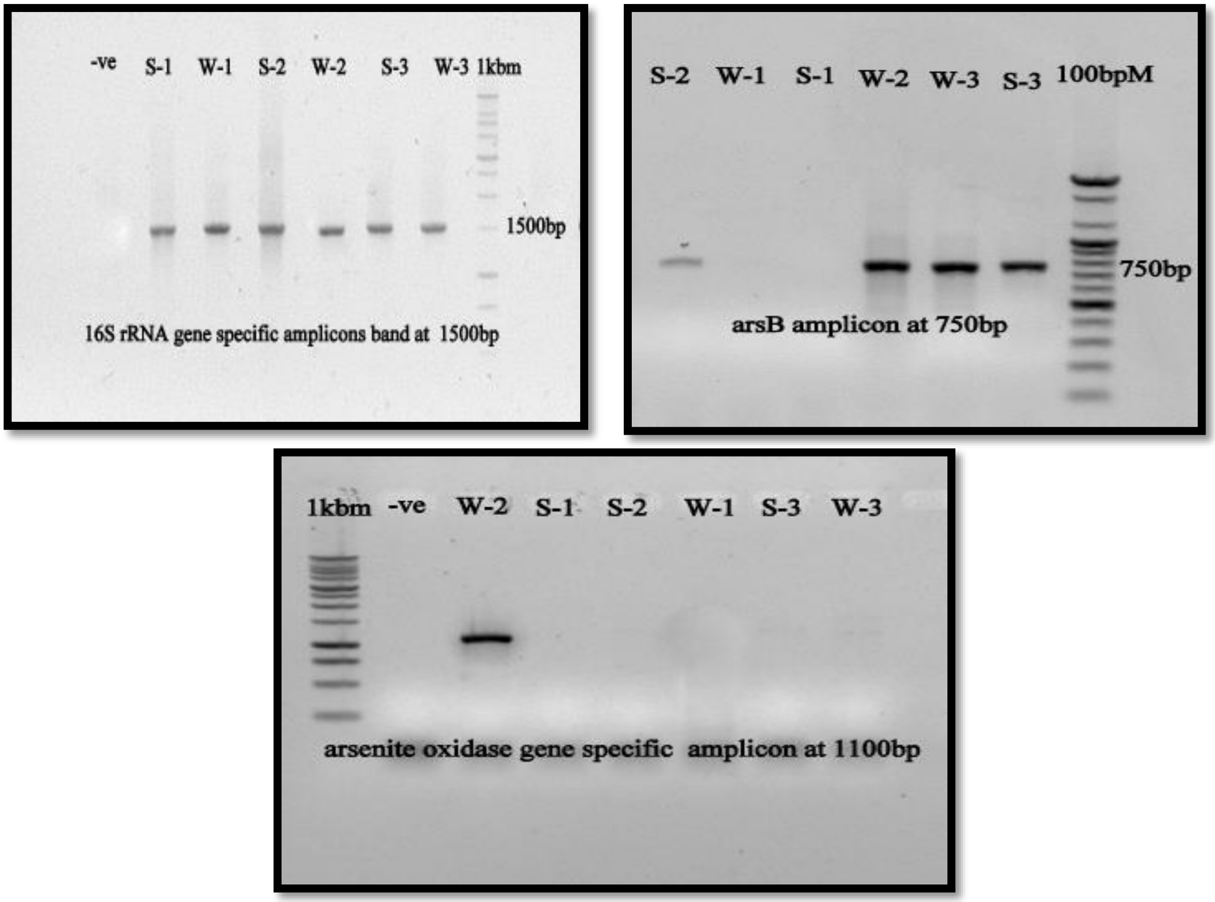

Figure S1:PCR specific amplicon of 16S rRNA (a), arsenite resistance gene *ars*B (b) and arsenite oxidizing gene *aox*B (c)of soil total DNA.

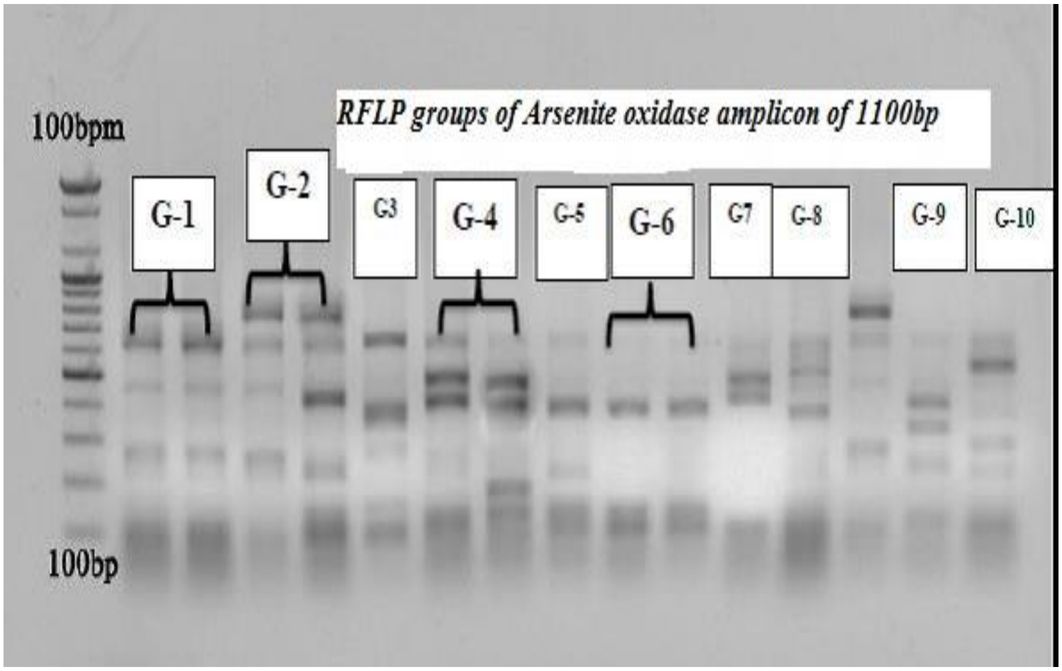

Figure S2: RFLP groups using *Alu*I restriction digestion of *aio*A gene containing clones.

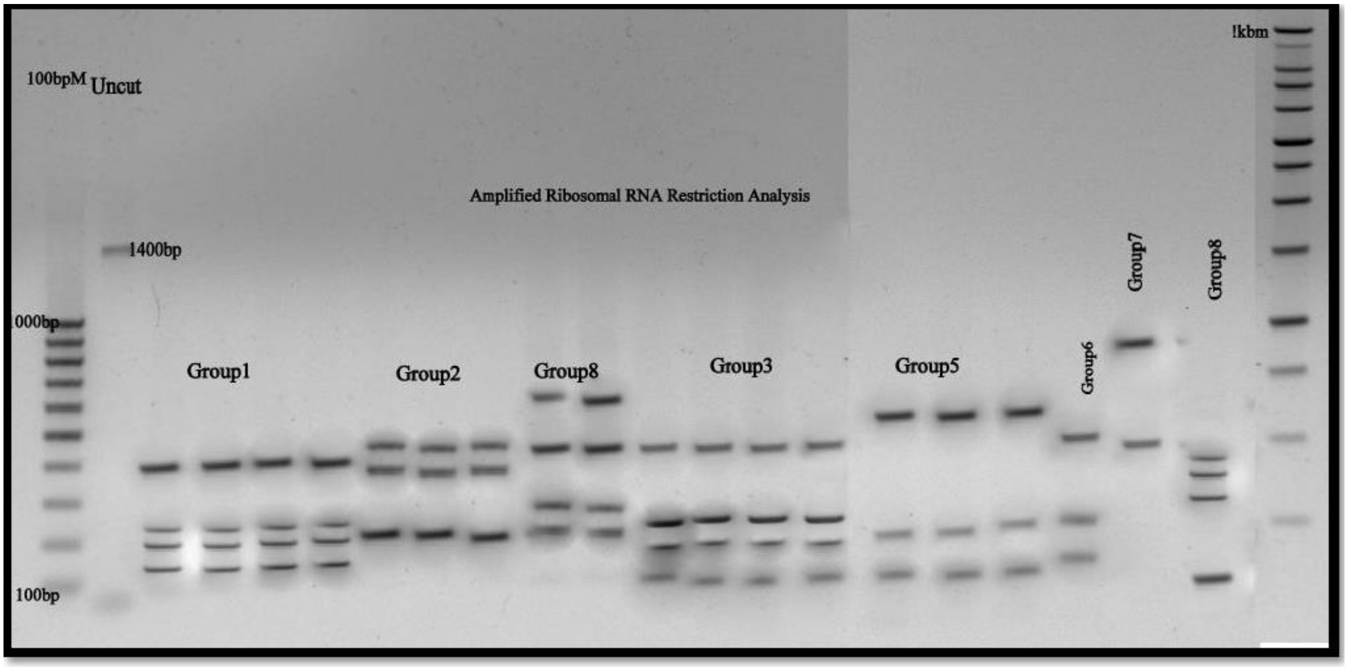

Fig. S3: Different fragments obtained from *Alu*1enzyme digestion of the 16S rRNA gene PCR product (approx. 1400-1450 bp) of arsenite resistant isolates. Representative groups are shown here. Uncut experimental DNA incubated under same condition was used as control. Marker used was 1 kb and 100 bp

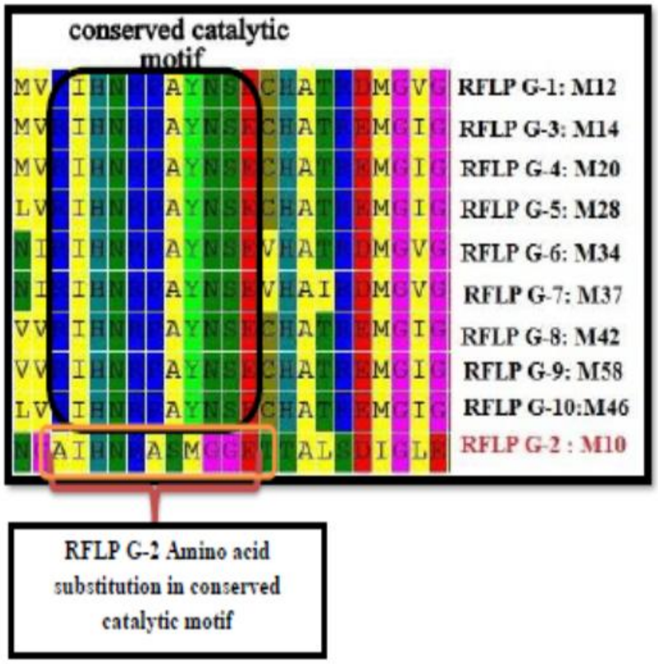

Fig.S4: Amino acid sequence alignment (MEGA 5) of ten RFLP groups representative cloned arsenite oxidase genes from WFDSL-2 soil sample showing conserved catalytic motif.

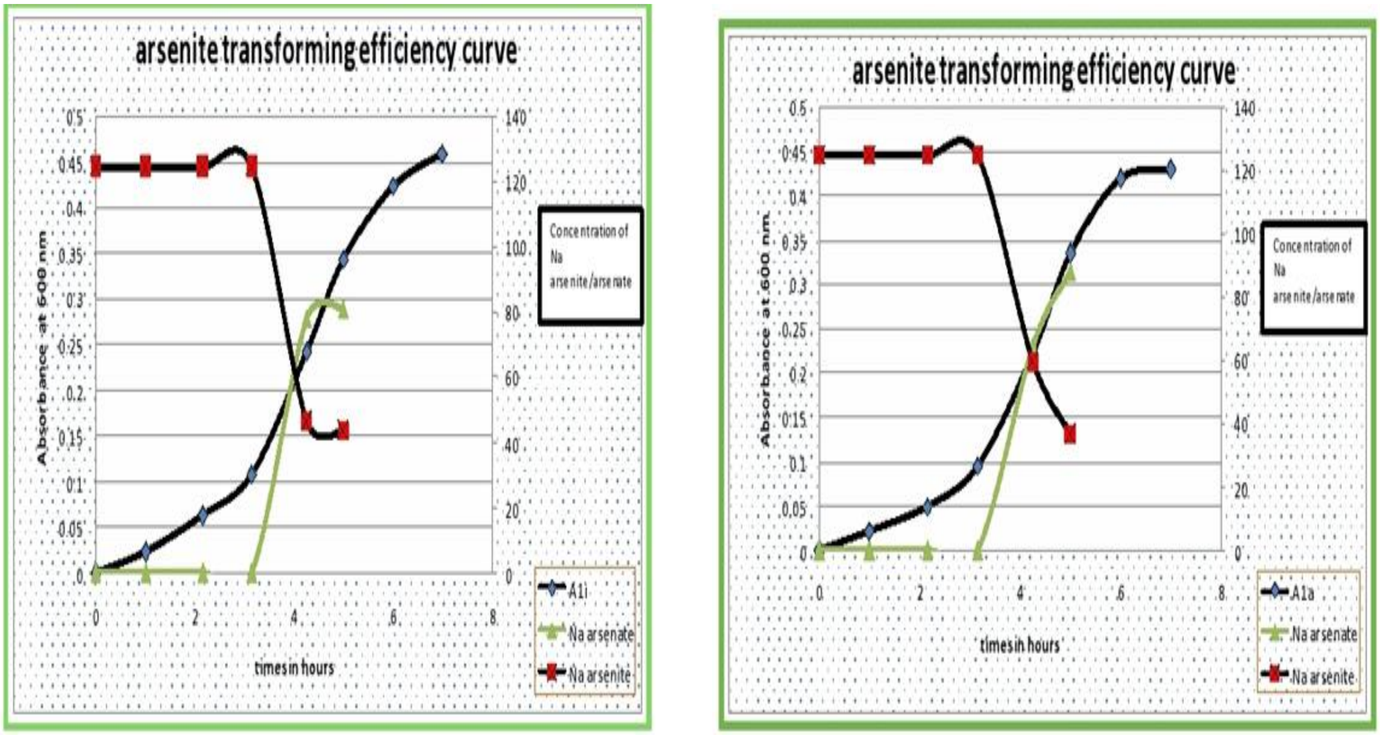

Fig. S5: Growth kinetics and corresponding oxidation of arsenite, As (III) to arsenate, As (V) by isolate (a) A1a (b) A1i
